# Subcutaneous delivery of mesenchymal stromal cells induces immunoregulatory effects in the lymph node prior to their apoptosis

**DOI:** 10.1186/s13287-024-04060-0

**Published:** 2024-11-17

**Authors:** Di Zheng, Tejasvini Bhuvan, Natalie L. Payne, Swee H. M. Pang, Senora Mendonca, Mark R. Hutchinson, Flyn McKinnirey, Charlotte Morgan, Graham Vesey, Laurence Meagher, Tracy S. P. Heng

**Affiliations:** 1 Department of Anatomy and Developmental Biology, Biomedicine Discovery Institute, Monash University, Clayton, VIC 3800 Australia; 2 School of Biomedicine, University of Adelaide, Adelaide, SA 5005 Australia; 3 Australian Research Council Centre of Excellence for Nanoscale BioPhotonics, University of Adelaide, Adelaide, SA 5005 Australia; 4 Regeneus Ltd, 2 Paddington Street, Paddington, NSW 2021 Australia; 5 Department of Materials Science and Engineering, Monash University, Clayton, VIC 3800 Australia; 6 Australian Research Council Training Centre for Cell and Tissue Engineering Technologies, Monash University, Clayton, VIC 3800 Australia

**Keywords:** Mesenchymal stem cells, Macrophages, Inflammation, Lymph nodes

## Abstract

**Background:**

Mesenchymal stromal cell (MSC) therapy commonly involves systemic infusion of MSCs, which undergo apoptosis in the lung and induce immunoregulatory macrophages that reduce disease. The relevance of this mode of action, however, is yet to be determined for MSCs administered via other routes. Here, we administered MSCs via subcutaneous (SC) injection into inflamed tissue and investigated the immunomodulatory effects on the local lymph node (LN), which is a major site for the initiation and regulation of immune responses.

**Methods:**

A mouse model of localised skin inflammation was established with low-dose lipopolysaccharide (LPS) to in vivo prime adipose-derived MSCs delivered via SC injection. We then analysed the immunomodulatory changes in the LN draining the inflamed tissue, as well as the neutrophil TNF response to LPS re-exposure.

**Results:**

When administered directly into the inflamed skin tissue, SC MSC injection induced an expansion of IL-10-producing MerTK^+^ subcapsular sinus macrophages and T cell zone macrophages, as well as activated CD44^+^ regulatory T cells (Tregs), in the draining LN, which was not observed in the non-draining LN. SC injection of viable, but not apoptotic, MSCs dampened TNF production by inflammatory cells in the draining LN when re-exposed to the inflammatory stimulus. SC injection of MSCs remote to the site of inflammation also did not attenuate the LN response to subsequent inflammatory challenge.

**Conclusions:**

MSCs delivered directly into inflamed skin activated immunoregulatory cells in the local LN and inhibited LN responsiveness to subsequent inflammatory challenge. The immunoregulatory effects of SC-injected MSCs in the LN require priming by inflammatory cytokines in the local milieu. Furthermore, SC-injected MSCs exert anti-inflammatory effects in the draining LN prior to their apoptosis, in contrast to intravenously delivered MSCs, where anti-inflammatory effects are linked to their apoptosis.

**Supplementary Information:**

The online version contains supplementary material available at 10.1186/s13287-024-04060-0.

## Introduction

Mesenchymal stromal cells (MSCs) possess therapeutic potential in treating various diseases, including myocardial infarction, graft-versus-host disease, and pulmonary diseases [1–4]. The therapeutic effects of MSCs have been attributed to their immunomodulatory capacity. The majority of studies have primarily focused on systemic infusion of MSCs via the intravenous (IV) route, which is the standard method for delivering various cell therapies [5–7]. However, IV-delivered cells get entrapped in the lung and are cleared quickly [7, 8]. Recent studies have shown that MSCs undergo apoptosis in the lung following systemic IV infusion and are efferocytosed by lung phagocytes, inducing an anti-inflammatory function in macrophages that underlies the therapeutic effects of MSC therapy [3, 8].

MSCs also produce soluble molecules (e.g. TGF-β, IDO, PGE2) and extracellular vesicles (collectively called the “secretome”) that have immunomodulatory properties [9, 10]. In some settings, the secretory properties of MSCs can be further utilised to provide sustained production of exogenous molecules for therapeutic effect [11–13]. Thus, for therapeutic applications that utilise the viable MSC secretome, the short dwell time of MSCs following systemic infusion is not ideal and would benefit from alternative methods of MSC delivery. Subcutaneous (SC) and intramuscular (IM) injections are two of the common methods of delivering MSCs directly to tissue and treating local pathologies [1, 6]. Studies have shown a longer dwell time of MSCs following SC and IM administration than IV infusion [5, 6].

Extravascular MSC treatment in some disease settings is preferable for reasons other than longer in vivo persistence. For example, when treating spinal cord injury or nerve injury, intrathecal injection of MSCs would bypass the blood-brain-barrier and increase the therapeutic effect at the disease site [14, 15]. In addition to local effects, remote and systemic therapeutic effects have also been reported with extravascular MSC treatment. IM administration of MSCs in the hamstring has been shown to improve cardiac function in xenogeneic animal models [2, 16, 17]. The demonstration of systemic and remote effects makes extravascular MSC treatment an attractive treatment option for certain diseases, as it can be less invasive.

Unlike IV-delivered MSCs, MSCs administered directly into the tissue are not expected to localise to the lung and become apoptotic. MSC apoptosis therefore may not play a predominant role in local MSC treatment. Furthermore, following local injection, the cellular fitness, in vivo persistence and, in turn, secretory properties, of MSCs are influenced by pro-inflammatory cytokines in the inflamed tissue [18]. As immune responses are initiated and maintained in the secondary lymphoid organs [19], the immunomodulatory responses generated by MSC treatment would involve resident immune cells, specifically those in the local lymph node (LN) draining the inflamed tissue. Yet, much remains to be known about the role of LN-resident cells in MSC treatment. In this study, we utilised a skin inflammation model to in vivo ‘prime’ MSCs and investigated the immunomodulatory changes in the draining LN induced by SC injection of viable versus apoptotic MSCs.

## Materials and methods

### Animals

Female 6-to-8-week-old C57BL/6 and BALB/c mice were obtained from Monash Animal Research Platform (MARP) and maintained under specific pathogen-free conditions at the Monash University Animal Research Laboratories (ARL). All animal experiments received approval from Monash University Animal Ethics Committee (Project ID: 23407) and were performed in accordance with the guidelines of the Australian Code of Practice for the Care and Use of Animals for Scientific Purposes. The work has been reported in line with the ARRIVE guidelines 2.0.

### Cell culture

Human MSCs were isolated from healthy donor lipoaspirate as previously described [4] (Monash human ethics approval #2007/1798; performed with informed patient consent). Prior to studies, MSCs were recovered from liquid nitrogen in MSC media (Table S1) and maintained in a 5% CO_2_, 37 °C humidified incubator for 24 h. After culture recovery, MSCs were harvested from culture flasks and filtered through 70 μm cell strainers (PC0349, Rowe Scientific) to avoid cell clumps. For co-culture assays, MSCs were resuspended at 6 × 10^5^ cells/ml in macrophage media (Table S1). For subcutaneous injection, MSCs were resuspended at 20 × 10^6^ cells/ml in Dulbecco’s PBS (D8537-500ML, Sigma-Aldrich).

### Apoptotic MSC preparation

Human adipose MSCs were treated with BH3-mimetic drugs, composed of 1.25 µM of BCL-2 inhibitor (ABT199), BCL-XL inhibitor (A1331852) and MCL-1 inhibitor (S63845), for 2.5 h to induce apoptosis, as previously described [8]. All cells were harvested after apoptosis induction, washed and labelled with Annexin V and propidium iodide (Table S2). Cells were confirmed to be in early apoptosis (Annexin V^+^PI^−^) via flow cytometry. Apoptotic MSCs were resuspended at 20 × 10^6^ cells/ml in Dulbecco’s PBS (D8537-500ML, Sigma-Aldrich) for in vivo application.

### Bioluminescent imaging

Human bone marrow MSCs were transduced with a bicistronic lentiviral vector encoding firefly luciferase and eGFP (fluc-GFP^+^ MSC), as previously described [4]. Prior to injection, fluc-GFP^+^ MSCs were culture recovered for 24 h and examined for their expression of fluc-GFP (~ 70%) via flow cytometry. Female 6-week-old BALB/c mice received 1 × 10^6^ fluc-GFP^+^ MSCs in Dulbecco’s PBS (D8537-500ML, Sigma-Aldrich) either via tail vein (IV), intraperitoneal (IP), or subcutaneous (SC) hock injection. In a separate experiment, mice received a subcutaneous injection of 1 × 10^6^ fluc-GFP^+^ MSCs 1 h after LPS injection either in the ipsilateral hock or the non-inflamed contralateral hock. Ten minutes prior to imaging, mice received an intraperitoneal injection of 200 µl of D-luciferin (150 mg/kg, VivoGlo, Promega). Mice were imaged under anaesthesia (2.5% isoflurane in oxygen), daily until the bioluminescence signal disappeared. Imaging was performed in an Ami-HTX (TrendBio) Small Animal Imager and analysed with Aura Imaging Software (Spectral Instruments Imaging). A region of interest (ROI) was manually drawn on each individual image around the MSC injection area. A value of radiance (photon/sec/cm^2^/sr) was generated by the software for each ROI, which was then standardised (subtracting the background value of uninjected mice) and plotted to compare the luminance.

### LPS-induced skin inflammation model

Female 6-week-old C57BL/6 mice were randomly assigned to untreated or LPS-treated groups. For LPS treatment, mice were lightly anaesthetised with inhaled isoflurane 1–5% /oxygen 1.5–2 L mix, distributed from an anaesthetic machine, and then received a subcutaneous injection of 30 ng LPS (50 µl of 600 ng/ml *Escherichia coli* O55:B5; tlrl-b5lps, InvivoGen) in the right hock. Mice then received viable or apoptotic MSCs (1 × 10^6^ in 50 µl) via subcutaneous injection 1 h after LPS injection either in the ipsilateral hock or the non-inflamed contralateral hock. Control mice received a subcutaneous injection of the same volume of Dulbecco’s PBS. For in vitro assays, popliteal LNs draining the injection sites were harvested 3 h after MSC injection. To analyse immunomodulatory changes in the LNs after SC MSC injection, mice received a subcutaneous injection of viable or apoptotic MSCs (1 × 10^6^ in 50 µl) in the ipsilateral or contralateral hock 4 h after LPS injection. Treated mice were monitored daily in accordance with ethics approval.

### Mouse skin homogenisation

Mice were euthanised by CO_2_ asphyxiation. Hair around the hock region was removed prior to tissue harvesting. The skin tissue around the hock region was carefully excised and harvested into a 2 mL chilled Eppendorf tube filled with 200 µl of tissue lysis buffer (150 mM NaCl, 15 mM Tris, 1% Triton X-100 and 1x Protease inhibitors (P2714, Sigma- Aldrich)). Then, tissue was homogenised with an electronic homogeniser Bio-Gen PRO200 (Pro Scientific) for three rounds of 15 s processing. Homogenised tissue was incubated on ice for 30 min with agitation, followed with centrifugation at 10,000 *g* for 10 min. The supernatant was harvested for cytokine analysis with Cytometric Bead Array assay (BD Biosciences). Pierce™ BCA protein assay kit (23225, ThermoFisher Scientific) was used to measure the total protein concentration in the skin homogenate and standardise the cytokine readouts.

### Mouse lymph node digestion

Popliteal LNs were harvested in RPMI and subjected to three rounds of enzymatic digestion in LN digestion media (Table S1), as previously described [20, 21]. In brief, LNs were incubated in LN digestion media at 37 °C followed by pipetting to dissociate cells from stroma mechanically. The digested cell suspension was collected in tubes pre-filled with cold FACS buffer (Table S1). Cells were centrifugated at 300 *g*, 4 °C and filtered through 70 µm cell strainers for downstream analysis.

### LPS stimulation assay

LN cells were resuspended in RPMI media (Table S1) and plated at 1 × 10^5^ cells/well onto a 96-well low-attachment plate (CLS3474, Corning), and stimulated with 10 µg/ml LPS for 4 h. For unstimulated groups, cells were plated in RPMI media without LPS. Monensin solution (1000x) (420701, BioLegend) was added to the cells at the same time as LPS to capture intracellular cytokines. Cells were then washed with PBS and stained with Live/Dead Fixable Blue Dead Cell Stain (Table S2), followed by antibodies to surface markers (CD45-APC-Cy7, Ly6G-A700, Ly6C-BV711, CD11b-BV650, CD11c-BV421, MHC II-BV510, MerTK-PE-Cy7, F4/80-A594; Table S2). Cells were fixed and permeabilised with FoxP3/Transcription Factor Staining Buffer (00552300, eBioscience) prior to labelling with antibodies for intracellular cytokines (TNF-APC and IL-10-PE; Table S2) for flow cytometric analysis.

### In vitro T cell assay

CD4^+^CD25^−^ naïve T cells were isolated from C57BL/6 mouse splenocytes. CD45^+^Ly6G^+^SSC-A^hi^ neutrophils, CD11b^+^MerTk^+^ macrophages, Ly6C^lo/hi^CD11b^+^ monocytes and CD11c^+^MHCII^+^ dendritic cells (DCs) were isolated from digested LNs from MSC-injected or untreated mice. Cells were labelled with antibodies (splenocytes: CD4-FITC and CD25-APC; LN cells: CD45-APC-Cy7, Ly6G-A700, Ly6C-BV711, MHC II-BV510, CD11b-APC, CD11c-BV421 and MerTK-PE-Cy7) for 30 min at 4 °C in the dark, then washed in FACS buffer to remove excess antibodies. Cells were filtered through a 40 µm cell strainer and resuspended in FACS buffer containing 1x propidium iodide (Table S2) for FACSorting on a BD Influx Cell Sorter (BD Biosciences). Sorted naïve T cells were labelled with CellTrace Violet dye (C34557, ThermoFisher Scientific) and plated onto a 96-well round-bottom plate pre-coated with 1 µg/ml purified NA/LE hamster anti-mouse CD3 (Table S2). Myeloid cells and T cells were plated at 1:5 ratio (2 × 10^4^ myeloid cells: 1 × 10^5^ T cells). 2 µg/ml of purified NA/LE hamster anti-mouse CD28 (Table S2) and recombinant IL-2 were added, and the cells were incubated in a humified 37 °C incubator for 72 h. Cells were labelled with antibodies (CD4-FITC, CD44-biotin and FoxP3-PE; Table S2) for flow cytometric analysis and counting beads were included for enumeration purposes. T cell proliferation was indicated by dilution of CellTrace Violet dye and measured as division index using FlowJo™ v10.8 software (BD Biosciences).

### Flow cytometric analysis

LN cells were washed with PBS and incubated with Live/Dead Fixable Blue Dead Cell Stain (Table S2) for 30 min at room temperature in the dark. After incubation, cells were washed in FACS buffer to remove excess dye, then labelled with antibodies to surface markers (Table S2) pre-diluted in FACS buffer for 30 min at 4 °C in the dark. Following surface staining, cells were washed in FACS buffer to remove excess unbound antibodies and were then fixed and permeabilised with FoxP3/Transcription Factor Staining Buffer (00552300, eBioscience) for 30 min at 4 °C. Cells were then labelled with antibodies to intracellular markers (Table S2) pre-diluted in 1x Permeablisation Buffer (00552300, eBioscience). Excess antibodies were removed by washing with FACS buffer. Labelled samples were resuspended in FACS buffer and filtered through 70 μm filter mesh prior to flow cytometric analysis. Unstained, single stained and fluorescence-minus-one (FMO) samples were included for instrumental set-up and experimental analysis. All samples were acquired on a BD LSRFortessa X-20 Cell Analyzer (BD Biosciences). Data analysis was performed using FlowJo™ v10.8 software (BD Biosciences) and FlowLogic software (Inivai Technologies).

### Real-time qPCR

Total RNA was extracted from cell pellets of whole LNs, purified Ly6G^+^ neutrophils and CD64^+^ monocytes using a RNeasy micro kit (74004, Qiagen) and reverse-transcribed with iScript (1708840, Biorad). The Universal SYBR Green Master Mix (1725271, Biorad) was added along with cDNA and 0.2 µM primers (Table S3) for a quantitative PCR reaction volume of 10 µl. Real-time PCR was run on Roche Lightcycler 480 with following program: Denaturation 95 ℃, 30 s; Amplification cycle consisted of denaturation 95 ℃, 10 s, annealing 50 ℃, 60 s, and extension 72 ℃, 45 s, for maximum 40 cycles. The relative quantification of cytokine transcripts is calculated relative to the housekeeping genes (β-actin and GAPDH) according this equation: 2^-ddCt = 2^-(dCt of Sample – dCt of Control) = 2^-(Sample (Ct of cytokine gene – Ct of housekeeping gene) - Control (Ct of cytokine gene – Ct of housekeeping gene)).

### Statistical analysis

Data were analysed using Student *t*-test or One-way ANOVA followed by Tukey’s multiple comparison test using GraphPad Prism (version 9). All statistical analyses were plotted as mean ± standard error of mean (SEM) and levels of significances were represented as * *p* < 0.05, ** *p* < 0.01, *** *p* < 0.001, ns, not significant.

## Results

### MSCs administered via SC injection exhibit a longer dwell time at the injection site compared to other injection routes

**Fig. 1:**
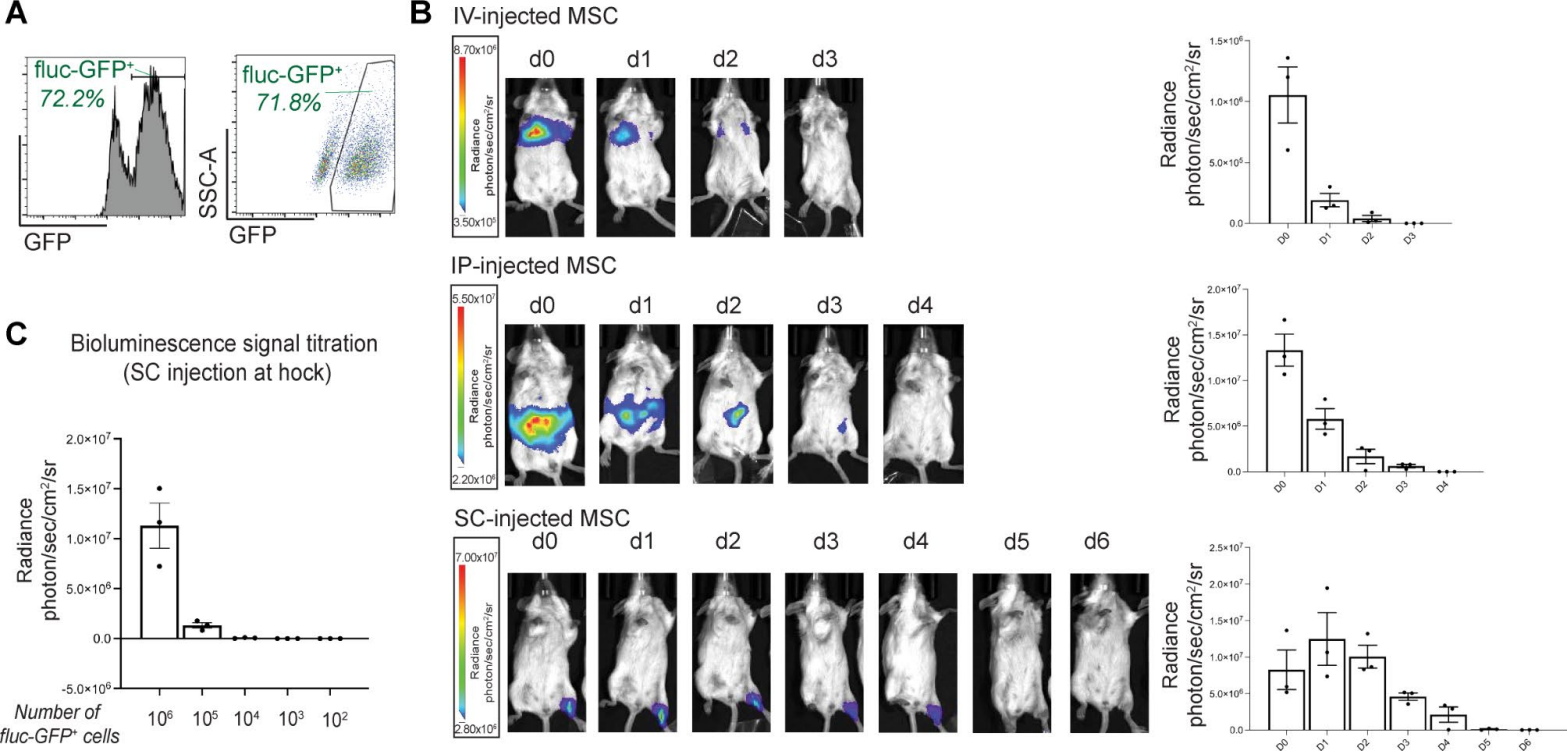
SC injected MSCs display a longer dwell time at the site of injection. (**A**), MSCs were transduced with a firefly luciferase (fluc) vector that also encodes eGFP (fluc-GFP) to enable the quantification of transduction efficiency (indicated by %flu-GFP^+^) by flow cytometry. The cell suspension prepared for injection was adjusted to ensure 1 × 10^6^ fluc-GFP^+^ MSCs per injection. (**B**) Representative bioluminescent images of luciferase-expressing MSCs after IV, IP and SC injections, with bar graphs present the changes in the radiance (*n* = 3 mice per group). (**C**) In vivo bioluminescent signal titration: various number of luciferase-expressing MSCs were injected via SC route into the hock to examine the lower threshold of the detection range. Data expressed as mean ± SEM; *n* = 3 mice per group

We first determined the in vivo persistence of MSCs delivered via SC injection compared to other injection routes. Bioluminescence imaging (BLI) was performed on healthy mice following IV, IP or SC injection of firefly luciferase-expressing MSCs (fluc-GFP^+^ MSC). The transduction efficiency of MSCs was quantified by flow cytometry indicated by the proportion of GFP-expressing cells (Fig. 1A, ~ 72%). The total number of cells to be injected was adjusted accordingly to ensure each mouse received 1 × 10^6^ fluc-GFP^+^ MSCs per injection.

MSCs administered via the IV route undergo apoptosis in the lung, a process shown to be necessary for inducing anti-inflammatory effects [8]. As expected, IV-injected MSCs were detected in the lung immediately after injection, and a rapid decay of their bioluminescent signals was observed within 1 day, with complete clearance on day 3 (Fig. 1B). IP-injected MSCs remained in the peritoneum around the injection site and the signals persisted for a slightly longer period of time than IV-injected MSCs, with complete clearance on day 4 (Fig. 1B). Compared to the IV or IP injection groups, MSCs administered via the SC route into the hock displayed the longest in vivo dwell time, with complete clearance on day 5 (Fig. 1B). SC-injected MSCs remained locally at the injection site (hock), without evidence of migration away from the injection site.

A titration of SC fluc-GFP^+^ MSC injection dose demonstrated the lower threshold for the BLI detection range being 10,000 fluc-GFP^+^ MSCs (Fig. 1C). Hence, the gradual disappearance of BLI signals at the SC injection site may indicate clearance of exogenous MSCs at the injection site after 5 days, or a potential dissemination of less than 10,000 cells to elsewhere in the body which could not be effectively detected.

### SC MSC injection into inflamed skin expands immunoregulatory IL-10^+^ macrophages in the draining LN

In inflammation and other disease states, immune responses are initiated and regulated in secondary lymphoid organs such as LNs. Studies characterizing the LN response to an acute inflammatory stimulus have commonly examined the popliteal lymph node draining the footpad where the inflammatory stimulus was injected [22]. The popliteal lymph nodes in mice are very small but easily located and separated from the skin and fascia [23]. Therefore, having confirmed that SC-injected MSCs remained at the injection site over a longer duration than IV- or IP-injected MSCs, we next established a localised acute inflammation model to provide an inflammatory microenvironment for SC-injected MSCs that would enable us to analyse the draining popliteal LN response.

**Fig. 2:**
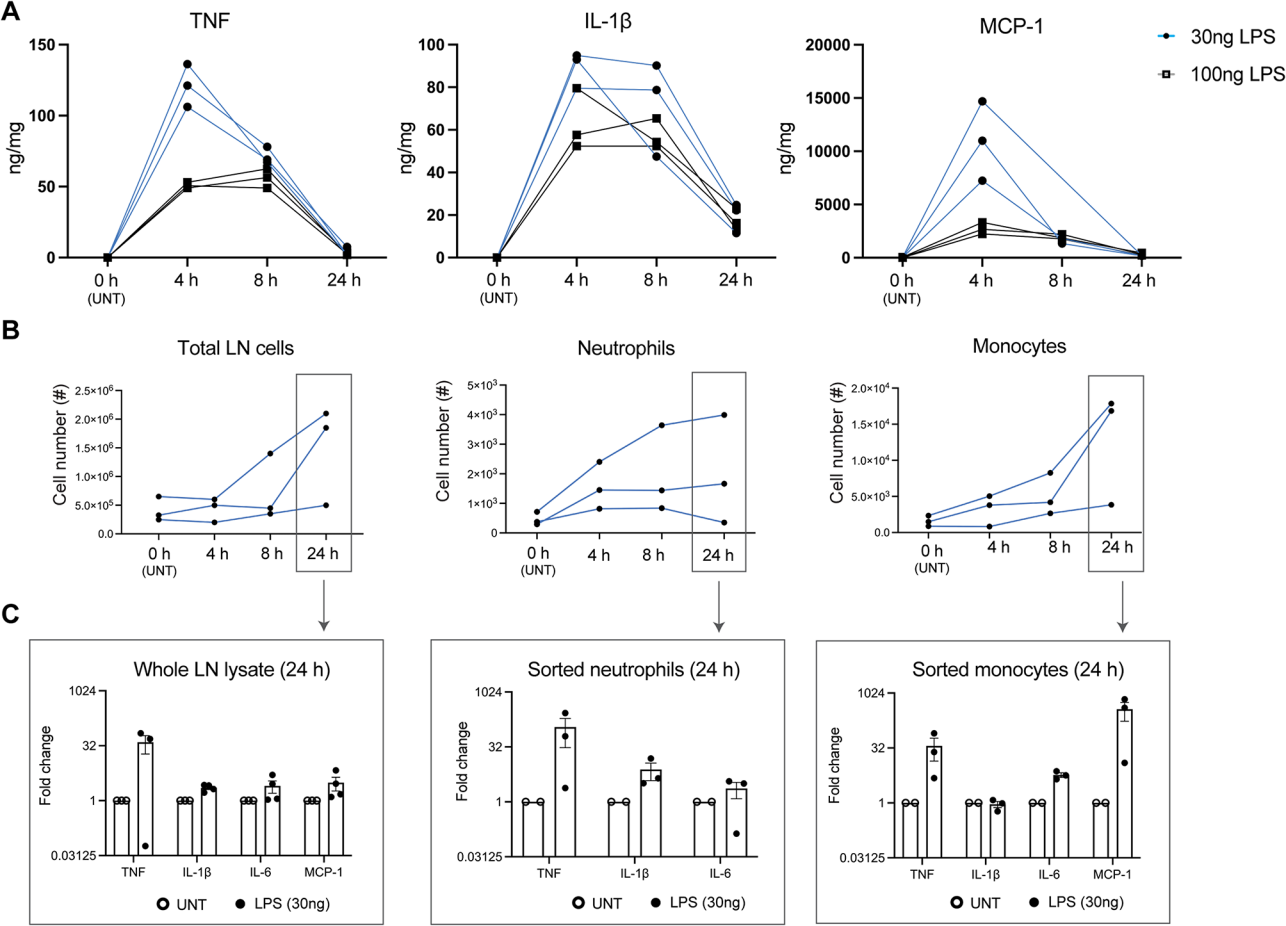
Establishing an acute inflammation model with low-dose LPS. (**A**) C57BL/6 mice were SC-injected with 30 ng or 100 ng LPS in 50 µl PBS in the left hock. At 0, 4, 8, 24 h post-LPS injection, skin tissue around the LPS injection site was excised and homogenised for cytokine analysis. Changes in pro-inflammatory TNF, IL-1β and MCP-1 in the skin tissues over the time course. *n* = 3 mice per timepoint from one experiment. (**B**) Draining LNs were harvested from mice at 0, 4, 8, 24 h after SC injection of 30 ng LPS in 50 µl PBS in the left hock. Total LN, neutrophil and monocyte cellularity at various timepoints post-LPS injection. (**C**) Inflammatory gene expression in whole LN cell lysate and sorted LN neutrophils and monocytes at 24 h post-LPS injection. Fold-change differences in mRNA expression of inflammatory genes were plotted after normalising mRNA expression level to housekeeping genes (GAPDH and β-actin). Data expressed as mean ± SEM; UNT (untreated LN) *n* = 2–3 mice per timepoint; LPS (LPS dLN) *n* = 3–4 mice per timepoint

TLR4 signalling pathway has been shown to be involved in establishing inflammatory hyperalgesia via the induction of pro-inflammatory cytokines such as TNF and IL-1β [24]. In a previous study, 100 ng of LPS (a TLR4 agonist) induced pro-inflammatory cytokines in the paw skin and inflammatory pain in the paw [24]. Due to institutional ethical constraints around animal welfare, we injected LPS into the hock, which is a non-weight bearing structure that drains to the same lymph node as the footpad, as a humane alternative to footpad injections [25]. We found 30 ng LPS injected into the hock was sufficient in inducing an acute inflammatory response in the skin, indicated by TNF, IL-1β and MCP-1 production, which peaked at 4 h post-LPS injection and resolved by 24 h (Fig. 2A), with concurrent expansion in total cellularity of the draining popliteal LN, increases in neutrophil and monocyte cell numbers from 4 h to 24 h (Fig. 2B) and elevation in their pro-inflammatory gene expression (TNF, IL-1β, IL-6 and MCP-1) (Fig. 2C). Surprisingly, a higher dose of LPS (100 ng) had a lower effect on cytokine production (Fig. 2A), possibly because higher doses of LPS induced a quicker inflammatory response that peaked at an earlier timepoint that was not captured here [24].

**Fig. 3:**
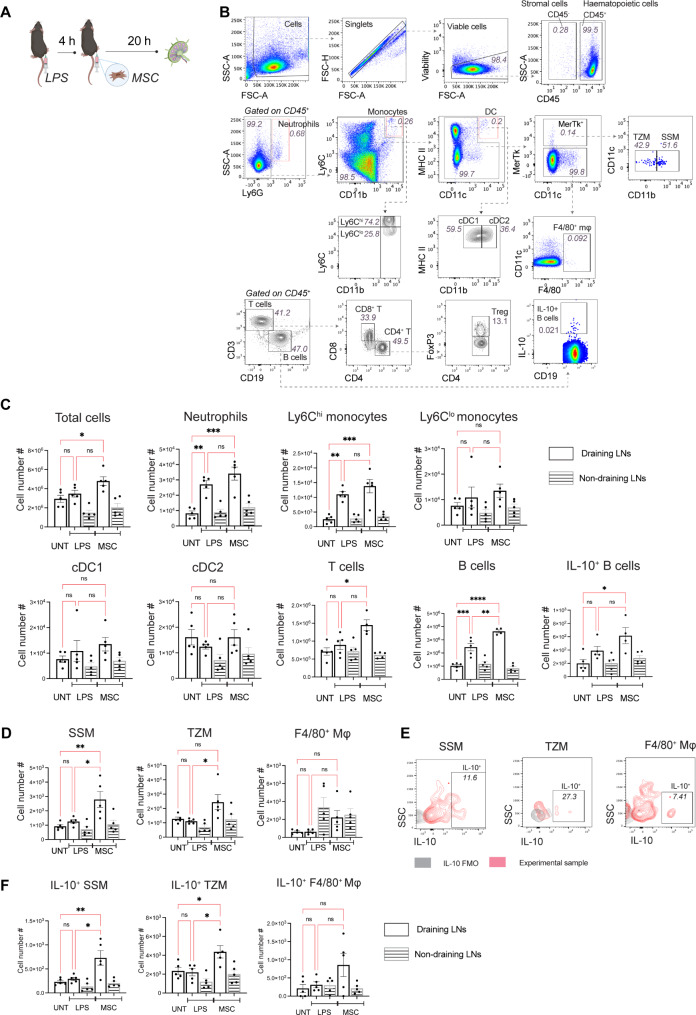
SC MSC injection expands IL-10-producing MerTK^+^ macrophages in the LN. (**A**) C57BL/6 mice were SC-injected with 30 ng LPS in 50 µl PBS in the left hock, and 4 h later 1 × 10^6^ human adipose MSCs in the same hock. Draining (dLN; white bar) and non-draining LNs (ndLN; shaded bar) were harvested 20 h after MSC injection for analysis. (**B**) Representative flow cytometry plots showing the gating strategies for delineating myeloid and lymphoid populations in the mouse LNs. (**C**) Changes in total cellularity and cell number of different immune populations in LNs following SC MSC injection. Data expressed as mean ± SEM; *n* = 4–5 mice per group, representative of 2 independent experiments. One-way ANOVA, Tukey’s multiple comparison, comparing UNT, LPS and MSC dLNs; * *p* < 0.05, ** *p* < 0.01, *** *p* < 0.001, ns, not significant. (**D**) Changes in cell number of different LN-resident macrophage subpopulations following MSC treatment. (**E**) Representative flow cytometry plots showing the identification of IL-10^+^ populations in LN macrophage subpopulations. (**F**) Changes in IL-10^+^ populations in LN macrophage subpopulations after LPS and SC MSC injection. Data expressed as mean ± SEM; *n* = 5 mice per group, representative of 2 independent experiments. One-way ANOVA, Tukey’s multiple comparison, comparing UNT, LPS and MSC dLNs; * *p* < 0.05, ** *p* < 0.01, *** *p* < 0.001, ns, not significant

Adipose tissue-derived MSCs were then delivered via SC injection into the inflamed tissue for in vivo priming with TNF, IL-1β and MCP-1 (which are cross-reactive between mouse and human cells [26]) and the popliteal LN analysed at 24 h (Fig. 3A, B) when the SC-injected MSCs were still detectable at the site of injection (Fig. 1B). Although neutrophils and monocytes in the LN expanded in number in response to LPS injection and were the main producers of pro-inflammatory cytokines in this model (Fig. 2C), SC MSC injection did not affect the cellularity of these populations, nor the number of DCs or T cells (Fig. 3C). There was, however, a significant increase in B cells in the LN following SC injection of MSCs into the inflamed tissue (Fig. 3C, white bar), which was not found in the non-draining popliteal LN (LN from the non-inflamed hock; Fig. 3C, shaded bar). Regulatory B cells (Bregs) have been reported to attenuate inflammation and are characterised by their ability to produce IL-10 ^27, 28, 29, 30^. We examined IL-10 production in B cells and found no increase in IL-10^+^ B cells (Fig. 3C), suggesting that SC MSC injection expanded the bulk B cell population rather than a specific regulatory B cell subtype.

In the lymph node, resident macrophages play crucial roles in immune regulation. Subcapsular sinus macrophages (SSM) act as gatekeepers by capturing foreign antigens in the lymph, whilst medullary sinus macrophages (MSM), medullary cord macrophages (MCM), tingible body macrophages and T cell zone macrophages (TZM) actively clear apoptotic cell debris, contributing to tissue homeostasis [19]. Following SC MSC injection, there was also an increase in MerTK^+^ SSM and TZM, but not F4/80^+^ MSM and MCM (Fig. 3D, white bar). None of these cellularity changes in response to LPS and MSC injection was observed in the non-draining popliteal LN (Fig. 3D, shaded bar).

LN-resident macrophages possess immunoregulatory function that protect against pathogenic infection and inflammatory insults [31, 32], and MerTk signalling in macrophages has been linked to anti-inflammatory outcomes [33, 34]. IL-10 has also been shown to shift macrophages toward an immunoregulatory and tolerogenic phenotype [35]. We therefore investigated whether the expansion of MerTK^+^ LN macrophages after SC MSC injection is associated with anti-inflammatory IL-10 production (Fig. 3E). The number of IL-10-producing macrophages remained unchanged in LPS mice, but SC MSC injection increased the number of IL-10-producing SSM and TZM in the draining popliteal LN (Fig. 3F, white bar). This increase was not observed in F4/80^+^ macrophages (Fig. 3F). IL-10^+^ macrophages also remained unchanged in the non-draining LN (Fig. 3F, shaded bar). Thus, SC MSC injection induced an increase in IL-10-producing MerTK^+^ macrophages but not F4/80^+^ macrophages in the draining LN.

### SC MSC injection into inflamed skin expands activated Tregs with decreased PD-1 expression in the draining LN

**Fig. 4:**
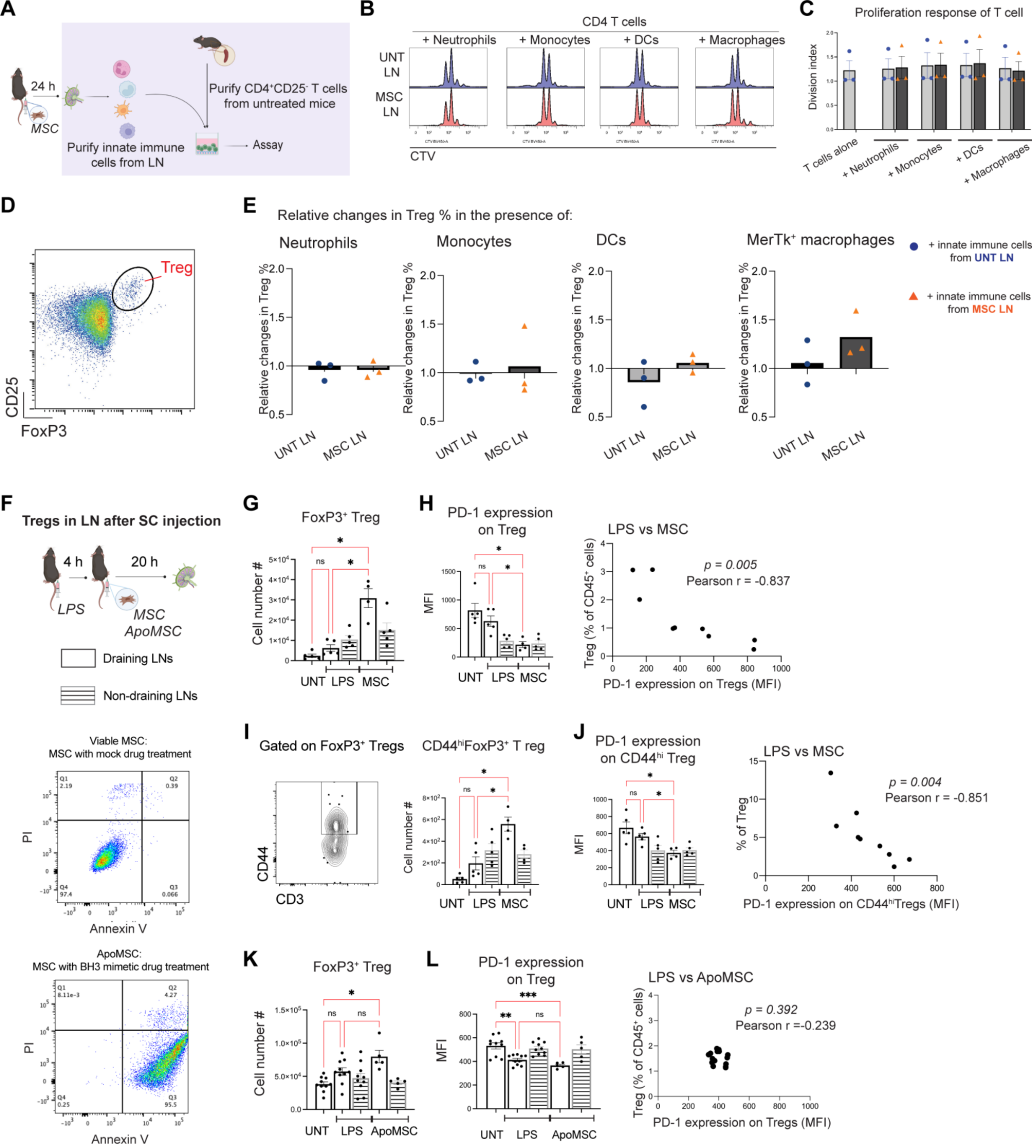
SC MSC injection expands activated Tregs in the LN. (**A**) CD45^+^Ly6G^+^SSC-A^hi^ neutrophils, Ly6C^lo/hi^CD11b^+^ monocytes, CD11c^+^MHCII^+^ DCs and CD11b^+^MerTk^+^ macrophages were purified from the LNs 24 h after SC MSC injection, and then added to CTV-labelled, anti-CD3/anti-CD28 activated splenic CD4^+^CD25^−^ T cells at 1:5 ratio for 72 h. (**B**) Flow cytometric profile of CTV dilution (indicating T cell proliferation) in the presence of innate immune cells purified from LN of untreated mice (UNT LN) or LNs from SC MSC-injected mice (MSC LN). (**C**) Division index of T cell proliferation. Data expressed as mean ± SEM; *n* = 3 mice per group. (**D**) Representative flow cytometry plot showing Treg gating. (**E**) Relative change in Treg proportion in the presence of LN neutrophils, monocytes, DCs or macrophages after standardising to T cells alone group). Data expressed as mean ± SEM; *n* = 3 mice per group. (**F**) Draining (dLN; white bar) and non-draining LNs (ndLN; shaded bar) were harvested from mice that received SC injection of viable or apoptotic MSCs into the inflamed hock. Flow cytometry plots showing viability of MSCs treated with DMSO (mock) or BH3 mimetics prior to SC injection. (**G**) Treg cellularity in the dLN (white bars) and ndLNs (shaded bars) following LPS and SC MSC injection. (**H**) Changes in the PD-1 expression on Tregs after SC MSC injection, and Pearson correlation analysis of Treg PD-1 expression level and Treg proportion in the dLNs from LPS and MSC groups (Pearson *r*=-0.861, *p* = 0.00137). (**I**) CD44 expression on activated Tregs, and changes in CD44^hi^ Tregs in dLNs and ndLNs after SC MSC injection. (**J**) Changes in PD-1 expression on CD44^hi^Tregs after SC MSC injection, and Pearson correlation analysis of CD44^hi^ Treg PD-1 expression level and Treg proportion in the dLNs from LPS and MSC groups (Pearson *r*=-0.883, *p* = 0.001). Data expressed as mean ± SEM; *n* = 5 mice per group. One-way ANOVA, Tukey’s multiple comparison, comparing UNT, LPS and MSC dLNs; * *p* < 0.05, ** *p* < 0.01, *** *p* < 0.001, ns, not significant. (**K**) Treg cellularity in dLNs and ndLNs following SC injection with ApoMSC. (**L**) Changes in PD-1 expression on Tregs after SC injection with ApoMSC, and Pearson correlation analysis of Treg PD-1 expression level and Treg proportion in the dLNs from LPS and ApoMSC groups (Pearson *r*=-0.239, *p* = 0.392). Data expressed as mean ± SEM; *n* = 5–10 mice per group. One-way ANOVA, Tukey’s multiple comparison, comparing UNT, LPS and ApoMSC dLNs; * *p* < 0.05, ** *p* < 0.01, *** *p* < 0.001, ns, not significant

Following IV administration of MSCs, MSC-primed monocytes/macrophages have been shown to enhance the differentiation of T cells to Tregs [7, 36, 37], which have crucial immunoregulatory function in the LN [38–40]. The increase in IL-10^+^ MerTK^+^ macrophages in the LN after SC MSC injection prompted us to examine the immunomodulatory capacity of macrophages following SC MSC injection. MerTK^+^ macrophages (SSM and TZM), neutrophils, monocytes and DCs were isolated from the LN of MSC-treated mice and co-cultured with CTV-labelled CD4^+^CD25^−^ T cells that were activated with anti-CD3/anti-CD28 (Fig. 4A). Activated T cells underwent proliferation, as indicated by CTV dilution (Fig. 4B). Neither macrophages, neutrophils, monocytes nor DCs suppressed T cell proliferation (Fig. 4C). However, analysis of Foxp3^+^ Tregs in the co-culture (Fig. 4D) revealed that macrophages from the LN of SC MSC-injected mice increased the proportion of Tregs, compared to macrophages from untreated mice (Fig. 4E). The expansion of Tregs was not observed with neutrophils, monocytes or DCs from the same LN of SC MSC-injected mice (Fig. 4E). Thus, MerTK^+^ macrophages from the LN of mice that received SC MSC injection exhibited an enhanced capacity to differentiate and/or expand Tregs.

As MerTK^+^ macrophages from the LN of SC MSC-injected mice displayed increased IL-10 production and enhanced capacity for Treg expansion, we performed a more detailed analysis of the Treg population within the LN (Fig. 4F). There was a pronounced increase in Foxp3^+^ Tregs in the draining popliteal LN of MSC-treated mice (Fig. 4G). Strikingly, Tregs from SC MSC-injected mice displayed decreased PD-1 expression (Fig. 4H). PD-1 expression on Tregs has been shown to be inversely proportional to their suppressive activity [41, 42]. A recent study showed that PD-1 on Tregs inhibits Treg activation and suppressive activity, as PD-1-deficient Tregs are more immunosuppressive [43]. PD-1–deficient Tregs exhibit an activated phenotype (CD44^hi^ CD62L^lo^) [43]. In line with this, there was an increase in activated CD44^hi^ Tregs (Fig. 4I), which displayed decreased PD-1 expression levels (Fig. 4J), in the LN of SC MSC-injected mice. No significant difference in Tregs was observed in the non-draining LN of SC MSC-injected mice.

As the changes in Tregs were observed in the draining LN when the bulk of SC-injected MSCs were still viable at the site of injection (indicated by strong bioluminescent signals) 20 h after injection, we next investigated whether these changes were also induced by SC-injection of apoptotic MSCs (ApoMSCs). MSCs were treated with BH3-mimetic drugs to induce apoptosis via the intrinsic pathway [8] and mice received a SC injection of ApoMSCs in early apoptosis (Annexin V^+^PI^−^) directly in the inflamed hock. No changes in the number of Tregs (Fig. 4K), or their PD-1 expression (Fig. 4L), were observed in the LN following SC injection of ApoMSCs at this early timepoint. Thus, SC injection of viable MSCs but not ApoMSCs increased the number of activated CD44^hi^ Tregs with decreased PD-1 expression in the draining LN.

### SC MSC injection into inflamed skin inhibits the TNF response of LN neutrophils to LPS rechallenge

**Fig. 5:**
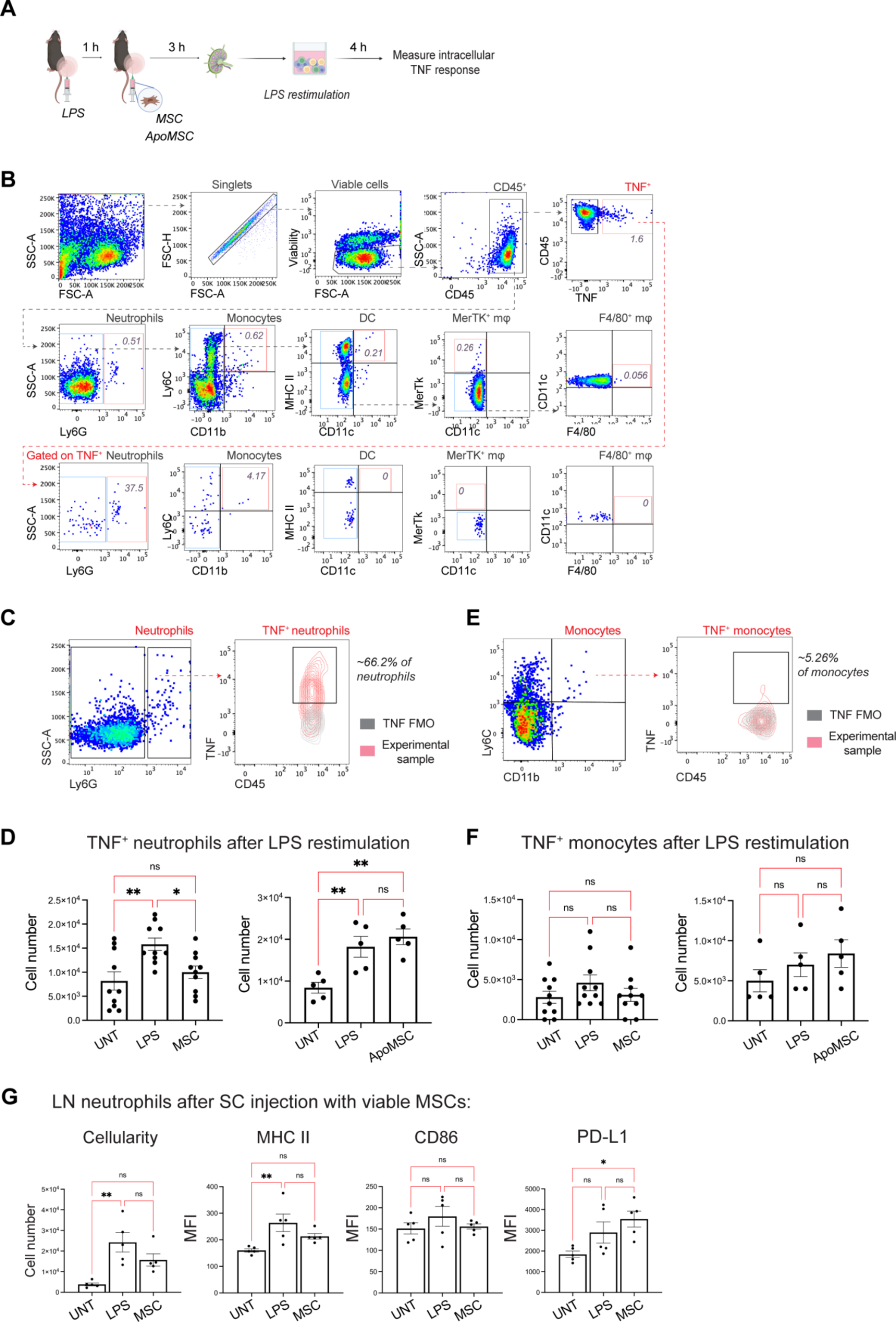
SC MSC injection inhibits TNF production by LN neutrophils in response to LPS re-exposure. (**A**) C57BL/6 mice were SC-injected with 30 ng LPS in 50 µl PBS in the left hock, and 1 h later 1 × 10^6^ human adipose MSCs in the same hock. The popliteal LN draining the LPS-injected hock was harvested 4 h after LPS injection (i.e. 3 h after MSC treatment) and restimulated with 10 µg/ml LPS for 4 h in vitro. Intracellular TNF production was used as an indicator of the pro-inflammatory response to the LPS rechallenge. (**B**) Flow cytometry plots showing the gating strategies for identifying the TNF-producing (TNF^+^) populations after in vitro restimulation of bulk LN cells with LPS. (**C**) Representative flow cytometry plots showing the gating for TNF^+^ neutrophils. (**D**) Number of TNF^+^ neutrophils after LPS restimulation of LN cells from mice that received SC injection of viable MSCs (left panel) or ApoMSC (right panel). Data expressed as mean ± SEM; *n* = 5–10 mice per group from 2 independent experiments. One-way ANOVA, Tukey’s multiple comparison; * *p* < 0.05, ** *p* < 0.01, *** *p* < 0.001, ns, not significant. E) Representative flow cytometry plots showing the gating for TNF^+^ monocytes. (**F**) Number of TNF^+^ monocytes after LPS restimulation of LN from mice that received SC injection of viable MSCs (left panel) or ApoMSC (right panel). Data expressed as mean ± SEM; *n* = 5–10 mice per group from 2 independent experiments. One-way ANOVA, Tukey’s multiple comparison; * *p* < 0.05, ** *p* < 0.01, *** *p* < 0.001, ns, not significant. (**G**) Cellularity and expression of MHCII, CD86 and PD-L1 on neutrophils in the dLN after SC MSC injection. Data expressed as mean ± SEM; *n* = 5 mice per group. One-way ANOVA, Tukey’s multiple comparison; * *p* < 0.05, ** *p* < 0.01, *** *p* < 0.001, ns, not significant

Having found that SC MSC injection promoted immunoregulatory cells in the LN draining the inflamed tissue, we next examined whether there was a suppression of the inflammatory response by analysing the production of pro-inflammatory TNF by LN cells following SC MSC injection. As MSCs were detectable up to 5 days after SC injection (Fig. 1B), a group of mice was injected with ApoMSCs as comparison, and the popliteal LN draining the inflamed tissue was analysed 4 h after LPS injection (Fig. 5A, B). Neutrophils were robust producers of TNF in response to LPS restimulation, with neutrophils accounting for ~ 40% of TNF-producing cells in the draining LN (Fig. 5C). Over 60% of neutrophils stained positive for intracellular TNF production (Fig. 5D). The popliteal LN from mice injected with viable MSCs showed a reduction in TNF-producing neutrophils upon restimulation with LPS (Fig. 5E, left panel). The reduction in TNF^+^ neutrophils was not observed in LN from mice that received ApoMSC (Fig. 5E, right panel). There were no significant changes in TNF^+^ monocytes with either viable MSC or ApoMSC injection (Fig. 5F, G). Further examination of neutrophils in the LN showed that whilst LPS injection increased their cell number and MHC class II expression, there were no significant changes with SC MSC injection (Fig. 5H). Thus, SC MSC injection decreased the TNF response of LN neutrophils to LPS, which required MSCs to be viable at the time of injection. The reduction in TNF was not due to a decrease in the number, or activation, of neutrophils in the LN.

### Direct MSC injection into the inflamed skin is necessary for immunoregulatory effects in the LN

**Fig. 6:**
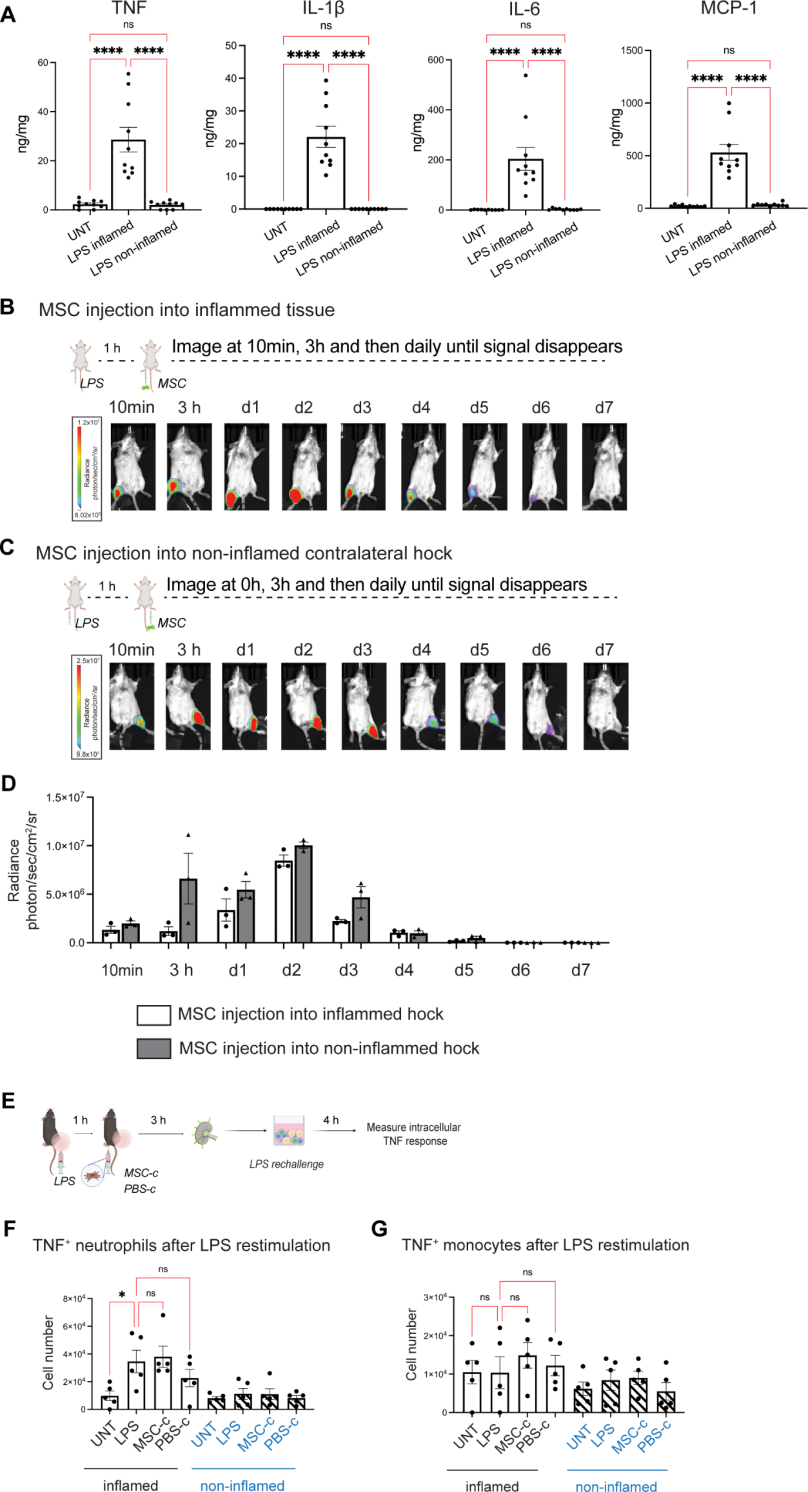
Contralateral SC MSC injection does not inhibit LN neutrophil TNF response to LPS re-exposure. (**A**) C57BL/6 mice were SC-injected with 30 ng LPS in 50 µl PBS in the left hock, and 4 h later the skin around the LPS injection site (LPS inflamed) and on the contralateral hock (LPS non-inflamed) was excised and homogenised for cytokine analysis. Data expressed as mean± SEM; *n* = 10 mice per group from 2 independent experiments. One-way ANOVA, Tukey’s multiple comparison, * *p* < 0.05, ** *p* < 0.01, *** *p* < 0.001, ns, not significant. (**B**) Bioluminescent imaging of mice that received SC injection of 30 ng LPS in 50 µl PBS in the left hock, and 1 h later 1 × 10^6^ luciferase-expressing MSCs in the same hock, or (**C**) in the non-inflamed hock on the contralateral side. (**D**) Bar graphs represent changes in radiance over time. *n* = 3 mice per group from one experiment. (**E**) C57BL/6 mice were SC-injected with 30 ng LPS in 50 µl PBS in the left hock. 1 h after LPS injection, mice received a SC injection of 1 × 10^6^ human adipose MSCs (MSC-c) or control PBS treatment (PBS-c) in the contralateral (non-inflamed) hock. The popliteal LNs draining the LPS-injected and the contralateral skin were harvested 4 h after LPS injection (i.e. 3 h after MSC-c/PBS-c treatment) and restimulated with 10 µg/ml LPS for 4 h. Intracellular TNF production was used as an indicator of the pro-inflammatory response to the LPS rechallenge. (**F**) Number of TNF^+^ neutrophils after LPS restimulation of LN cells from mice that received SC injection of MSC or control PBS on the contralateral hock. Data expressed as mean ± SEM; *n* = 5 mice per group. One-way ANOVA, Dunnett’s multiple comparison; * *p* < 0.05, ** *p* < 0.01, *** *p* < 0.001, ns, not significant. (**G**) Number of TNF^+^ monocytes after LPS restimulation of LN cells from mice that received SC injection of MSC or control PBS on the contralateral hock. Data expressed as mean ± SEM; *n* = 5 mice per group. One-way ANOVA, Dunnett’s multiple comparison; * *p* < 0.05, ** *p* < 0.01, *** *p* < 0.001, ns, not significant.

The immunomodulatory effects induced by SC MSC injection were observed in the LN draining the inflamed hock, but not in the non-draining LN. We therefore investigated whether SC MSC injection into the non-inflamed hock would induce the same effects. Using the above LPS-induced inflammation model, we first confirmed that LPS injection into the hock led to an increase in pro-inflammatory cytokines TNF, IL-1β, IL-6 and MCP-1 in the skin from the inflamed hock and did not induce any cytokine changes on the contralateral side (non-inflamed hock) (Fig. 6A). We then repeated the bioluminescence imaging to determine whether local inflammation affects the in vivo persistence of SC-injected MSCs. Fluc-GFP^+^ MSCs were injected via the SC route into the inflamed skin area (Fig. 6B) or the non-inflamed contralateral hock (Fig. 6C) 1 h post-LPS injection. Mice were imaged 10 min after SC MSC injection, 3 h later and then daily. Local inflammation did not increase the bioluminescent signals of fluc-GFP^+^ MSCs, neither did inflammation at a distance from MSC injection drive cell migration beyond the injection site (Fig. 6B, C). The bioluminescent signals lasted around 5 days, with a slight increase in bioluminescence signal during the first 2 days (Fig. 6D). Direct imaging of the popliteal LNs did not show drainage of significant numbers of MSCs to the local popliteal LN (data not shown). Although unlikely, we cannot rule out the potential dissemination of small numbers of MSCs, which were below the detection range, to other sites of the body.

We next investigated whether contralateral SC MSC injection into the non-inflamed hock could remotely regulate the inflammatory response to LPS within the draining LN (Fig. 6E). Analysis of the popliteal LN draining the inflamed hock 4 h after LPS injection showed that neither contralateral MSC injection nor PBS control treatment had an effect on the number of TNF-producing neutrophils or monocytes in response to LPS restimulation (Fig. 6F, G; inflamed). There was also no change in the number of TNF^+^ neutrophils or monocytes in the popliteal LN from the non-inflamed hock in any of the treatment groups (Fig. 6F, G; non-inflamed). Thus, in contrast to direct MSC injection into inflamed tissue, contralateral MSC injection did not affect the LN inflammatory response to LPS. This finding indicates that ‘priming’ of SC-injected MSCs by inflammatory cytokines in the local milieu is important for their immunoregulatory effects in the LN.

Taken together, our data demonstrate that SC injection of MSCs into inflamed tissue induced an increase in immunoregulatory cells in the tissue-draining LN and reduced the responsiveness of neutrophils to subsequent inflammatory challenge. In contrast to IV delivery of MSCs, the immunoregulatory effects of SC MSC injection on the local draining LN are not coupled with MSC apoptosis.

## Discussion

Effective clinical translation of MSC therapy has been hindered by several issues, including unclear mechanisms [44], variability in potency among MSC products and a lack of appropriate potency assays that focus on the immunomodulatory capacity of MSCs [45, 46]. Studies on IV-infused MSCs point to a role for MSC apoptosis in the lung and efferocytosis by lung phagocytes in the anti-inflammatory effects of MSC therapy [8]. However, MSCs administered via extravascular routes do not undergo apoptosis in the lung but still demonstrate therapeutic potential [6, 17]. In a localised acute inflammation model established with low-dose LPS, viable MSCs primed by inflammatory cytokines in the local milieu induced immunoregulatory cells in the local draining LN and inhibited the LN neutrophil response to inflammatory re-exposure. These effects occurred at an early timepoint where SC-injected MSCs were still viable, and were also not observed when ApoMSCs were injected. Thus, the immunomodulatory mechanisms of MSCs are affected by the injection route by which MSCs are delivered, which has implications for the development of MSC potency assays for clinical translation.

Our data confirmed that, unlike IV-injected MSCs, MSCs injected via the SC route do not migrate away from the injection site or undergo apoptosis in the lung. Signals of SC-injected MSCs did not persist at the site of injection beyond 5–6 days, regardless of whether local or remote tissue inflammation was present. Interestingly, we observed an increase in the MSC bioluminescence signals 1–2 days after MSC injection, which has also been reported previously by others to be due to hypoxia-induced promoter activation [47]. However, the fluc expression in our study is driven by a different promoter [4]. The transient increase in MSC signal is unlikely to be related to MSC proliferation as it was followed by a rapid loss of signal, which was not diffused to other tissues. Previous findings that MSC apoptosis can be induced by cytotoxic T cells and natural killer cells [3] or, in their absence, possibly myeloid cells with cytotoxic activity [8], indicate that interactions with immune cells affect the dwell time of MSCs in vivo. The differences in dwell time and biodistribution of IV- and SC-injected MSCs suggest different mechanisms by which the host immune responses are regulated in these two MSC delivery settings.

In this model, whereby SC-injected MSCs are primed by inflammatory cytokines within the injection site, neutrophils in the tissue-draining LN displayed a reduced capacity for TNF production that was not due to a decrease in neutrophil cell number or activation. This decrease in neutrophil response to inflammatory challenge was not seen in mice that received SC injection of ApoMSCs, indicating that MSCs must be viable at the time of injection. Whilst it is possible that SC-injected MSCs will exert local effects after undergoing some apoptosis in vivo, the sustained bioluminescence signals over the first 2 days indicate that the majority of MSCs were still viable at the early timepoint their immunomodulatory effects in the LN were measured, in contrast to IV-administered MSCs, whereby the host response to MSC apoptosis is essential [8]. The effects of SC-injected MSCs at this early timepoint were possibly mediated by the host immune response to the viable cellular product or its bioactive secretome. However, SC injection of MSCs in the contralateral, non-inflamed hock failed to mount a similar attenuated response towards inflammatory rechallenge. Whether the ineffectiveness of SC MSC injection at a distance from the inflamed tissue could be compensated by increasing the MSC dose requires further exploration. More likely, the loss of immunomodulatory effects was due to the absence of MSC priming by inflammatory cytokines.

Our data showed that the induction of IL-10 production in macrophages reported in IV and IP MSC treatment [48, 49] is also relevant in SC MSC treatment. When administered directly into the inflamed tissue, SC MSC injection induced immunoregulatory macrophages in the local LN draining the inflamed tissue. Interestingly, an expansion in IL-10-producing macrophages was observed only in the MerTK^+^ SSM and TZM populations, and not in the F4/80^+^ macrophage populations (MSM and MCM). This could be due to the F4/80^+^ macrophages residing deeper within the LN [32, 50] and are therefore less accessible. MerTK is an efferocytic receptor, and MerTK^+^ TZMs have been reported to have a role in clearing apoptotic cells and maintaining immune homeostasis and tolerance [51]. In addition, studies have demonstrated a role for MerTK signalling in attenuating inflammation [33, 34]. The inhibitory role of MerTK signalling pathway has been previously reported in an LPS-induced lung inflammation model [34]. Whether the selective expansion of IL-10-producing MerTK^+^ macrophages is associated with activation of MerTK-related immunomodulatory pathway should be investigated further.

The immunoregulatory function of MSC-‘primed’ MerTk^+^ macrophages was confirmed by the demonstration that LN macrophages, but not neutrophils, monocytes or DCs, from SC MSC-injected mice promoted Treg expansion in vitro. In the macrophage/T cell co-cultures, the starting naïve T cell population were CD4^+^CD25^−^ cells sorted from bulk splenocytes. Therefore, we could not exclude the possibility that the Foxp3^+^ Tregs arose by proliferation from a small population of CD25^−^ Tregs (< 1% of Tregs), rather than conversion from naïve T cells [52]. Importantly, SC MSC injection also induced an increase in activated Tregs in the inflammation-draining LN. The activated CD44^+^ Treg population displayed decreased PD-1 expression, which is an indicator of enhanced suppressive function in Tregs [43]. The increase in activated Tregs with decreased PD-1 expression was only observed in mice that received SC injection with viable MSCs, but not ApoMSCs. Thus, our findings showed that the induction of immunoregulatory macrophages is common across various routes of MSC delivery, and further established a requirement for viable MSCs in the induction of immunoregulatory cells in SC MSC treatment.

SC MSC injection also induced an expansion of B cells in the draining LN. IP MSC treatment in a colitis model has been shown to polarise macrophages to induce IL-10^+^ T and B cells [49]. Whilst in our model, the increase in B cells in the draining LN was not related to a change in IL-10-producing Bregs, we did not assess other markers associated with Breg function. Further investigation on the B cell response following SC MSC injection will be needed to determine whether the expansion post-MSC injection is related to immunoregulation or allorecognition [53].

## Conclusions

Our study demonstrated apoptosis-independent effects of MSCs in driving the immunoregulatory changes in the local LN. Specifically, SC-injected MSCs primed by inflammatory cytokines in the skin induce immunoregulatory cells in the local LN and reduce neutrophil responsiveness to inflammatory challenge. These effects contrasted with the apoptosis-induced immunomodulatory/anti-inflammatory effects of IV-injected MSCs, suggesting different injection routes affect the mode of action of MSCs. Therapeutic strategies towards more effective resolution of inflammation require a better understanding of context-dependent mechanisms of MSC treatment. Knowledge gained from this study has implications for the development of MSC potency assays for clinical translation.

## Electronic supplementary material

Below is the link to the electronic supplementary material.

Supplementary Material 1

## Data Availability

The data generated in this study are available within the article and its supplementary data files. All raw data are available upon request.

## Abbreviations

ApoMSC: apoptotic MSC
BLI: Bioluminescence Imaging
Breg: regulatory B cell
fluc-GFP: firefly luciferase-Green Fluorescent Protein
IM: Intramuscular
IP: Intraperitoneal
IV: Intravenous
LN: Lymph Node
LPS: Lipopolysaccharide
MCM: Medullary Cord Macrophage
MSC: Mesenchymal Stromal Cell
MSM: Medullary Sinus Macrophage
SC: Subcutaneous
SEM: Standard Error of Mean
SSM: Subcapsular Sinus Macrophage
Treg: regulatory T cell
TZM: T cell Zone Macrophage
