# Supplementary material for: Subcutaneous delivery of mesenchymal stromal cells induces immunoregulatory effects in the lymph node prior to their apoptosis
Source: Stem Cell Res Ther. 2024 Nov 17;15:432. doi: 10.1186/s13287-024-04060-0 (PMC11572146; doi:10.1186/s13287-024-04060-0)
Supplement: Supplementary file 1 — Supplementary Material 1 [file 13287_2024_4060_MOESM1_ESM.docx]

**Supplementary Material**

**Subcutaneous delivery of mesenchymal stromal cells induces immunoregulatory cells in the lymph node and protects against inflammatory rechallenge**

Di Zheng^1^, Tejasvini Bhuvan^1^, Natalie L. Payne^1^, Swee H.M. Pang^1^, Senora Mendonca^1^, Mark R. Hutchinson^2,3^, Flyn McKinnery^4^, Charlotte Morgan^4^, Graham Vesey^4^, Laurence Meagher^5,6^, Tracy S. P. Heng^1,6*^

^1^ Department of Anatomy and Developmental Biology, Biomedicine Discovery Institute, Monash University, Clayton, VIC 3800, Australia. ^2^ School of Biomedicine, University of Adelaide, Adelaide, SA 5005, Australia. ^3^Australian Research Council Centre of Excellence for Nanoscale BioPhotonics, University of Adelaide, Adelaide, SA 5005, Australia. ^4^ Regeneus Ltd, 2 Paddington Street, Paddington, NSW, 2021, Australia. ^5^ Department of Materials Science and Engineering, Monash University, Clayton, VIC 3800, Australia; ^6^ Australian Research Council Training Centre for Cell and Tissue Engineering Technologies, Monash University, Clayton, VIC 3800. *Address correspondence to: Tracy.Heng@monash.edu

**Supplementary Table 1: Media and buffers composition**

| Media/Buffer | Composition |
| --- | --- |
| MSC media | - aMEM (12561072, ThermoFisher Scientific) - 16.5% FBS (batch-tested, Gibco) - 100 U/ml penicillin-streptomycin (15140122, Gibco) - 1 % GlutaMAX Supplement (35050079, Gibco) |
| Macrophage media | - DMEM - 10% heat-inactivated FBS (batch-tested, Gibco) - 100U/ml penicillin-streptomycin (15140122, Gibco) - 1% GlutaMAX Supplement (35050079, Gibco) |
| RPMI media | - RPMI 1640 - 10% heat-inactivated FBS (batch-tested, Gibco) - 100U/ml penicillin-streptomycin (15140122, Gibco) - 1% GlutaMAX Supplement (35050079, Gibco) - 0.1mM b-mercaptoethanol (M3148, Sigma-Aldrich) |
| LN digestion media | - RPMI 1640 - 0.2mg/ml Collagenase P (11213865001, Roche) - 0.8mg/ml Dispase (17105041, Life technologies) - 0.1mg/ml DNase I (11284932001, Roche) |
| FACS buffer | - 1x PBS - 0.1% BSA (Sigma) - 5mM EDTA (15575020, ThermoFisher Scientific) |
| RBC lysis buffer | - RO water - 8.3mg/ml Ammonium Chloride - 10mM TrisHCL |
| EDTA buffer | - 1x PBS - 5mM EDTA (15575020, ThermoFisher Scientific) |

**Supplementary Table 2: Antibodies and dyes for flow cytometry analysis**

| Viability dye | | Fluorochrome | Catalogue number, Company |
| --- | --- | --- | --- |
| Live/Dead Fixable Blue Dead Cell Stain | | UV excitation | L23105, ThermoFisher Scientific |
| Propidium Iodide | |  | P4170-10MG, Sigma-Aldrich |
| Annexin V | | FITC | 556419, BD Biosciences |
| Antigen | **Clone** | **Fluorochrome** | **Catalogue number, Company** |
| CD45 | 30-F11 | FITC | 553080, BD Biosciences |
| CD45 | 30-F11 | APC-Cy7 | 557659, BD Biosciences |
| Ly6C | HK1.4 | BV711 | 128037, Biolegend |
| Ly6G | 1A8 | A700 | 561236, BD Biosciences |
| Ly6G | 1A8 | A594 | 127647, Biolegend |
| CD11b | M1/70 | APC | 50-0112-80, eBioscience |
| CD11b | M1/70 | BV650 | 563402, BD Biosciences |
| CD11c | N418 | FITC | 117306, Biolegend |
| CD11c | HL3 | PE | 557401, BD Biosciences |
| CD11c | N418 | BV421 | 117330, Biolegend |
| MerTK | DS5MMER | PE-Cy7 | 25-5751-82, invitrogen |
| F4/80 | BM8 | BV421 | 123124, Biolegend |
| F4/80 | BM8 | A594 | 123140, Biolegend |
| CD3e | 145-2C11 | Pacific Blue | 562600, BD Biosciences |
| CD3e | 17A2 | BV510 | 100234, Biolegend |
| CD3e | 17A2 | Biotin | 100244, Biolegend |
| CD3e | 145-2C11 | Purified NA/LE | 553057, BD Biosciences |
| CD4 | GK1.5 | FITC | 553729, BD Biosciences |
| CD4 | RM4-5 | A700 | 100536, Biolegend |
| CD8 | 53-6.7 | Pacific Blue | 100725, Biolegend |
| CD25 | PC61 | APC | 102012, Biolegend |
| CD28 | 37.51 | Purified NA/LE | 553294 BD Biosciences |
| CD44 | IM7 | Biotin | 553132, BD Biosciences |
| MHC II | M5/114.15.2 | BV510 | 107635, Biolegend |
| PD-1 | J43 | PerCP-Cy5.5 | 46-9985-82, Invitrogen |
| IL-10 | JES5-16E3 | PE | 12-7101-82, Invitrogen |
| IL-10 | JES5-16E3 | APC | 554468, BD Biosciences |
| TNF | MP6-XT22 | FITC | 554418, BD Biosciences |
| TNF | MP6-XT22 | APC | 554420, BD Biosciences |
| FoxP3 | FJK-16s | PE | 12-5773-82, eBioscience |
| Streptavidin |  | PE-Cy7 | 557598, BD Biosciences |
| Streptavidin |  | PerCP-Cy5.5 | 551419, BD Biosciences |
| Streptavidin |  | BV605 | 563260, BD Biosciences |
| Streptavidin |  | BV786 | 563858, BD Biosciences |

**Supplementary Table 3: Primer sequences for quantitative PCR analysis**

|  | Forward | Reverse |
| --- | --- | --- |
| Mouse β-actin | 5′-AGGCATCCTCACCCTGAAGTA | 5′-CACACGCAGCTCATTGTAGA |
| Mouse GAPDH | 5′-TGACCTCAACTACATGGTCTACA | 5′-CTTCCCATTCTCGGCCTTG |
| Mouse TNF | 5′-GCCTCTTCTCATTCCTGCTTG | 5′-CTGATGAGAGGGAGGCCATT |
| Mouse IL-1β | 5′-AACCTGCTGGTGTGTGACGTTC | 5′-CAGCACGAGGCTTTTTTGTTGT |
| Mouse MCP-1 | 5′-CCACTCACCTGCTGCTACTCAT | 5′-TGGTGATCCTCTTGTAGCTCTCC |
| Mouse IL-6 | 5′-ACAACCACGGCCTTCCCTACTT | 5′-CACGATTTCCCAGAGAACATGTG |
